# Neurosyphilis With Concomitant Respiratory Failure: A Case Study and Treatment Considerations

**DOI:** 10.7759/cureus.41363

**Published:** 2023-07-04

**Authors:** Karlyle Bistas, Maheen Mirza

**Affiliations:** 1 Psychiatry and Behavioral Sciences, Wayne State University Detroit Medical Center, Detroit, USA; 2 School of Medicine, Medical University of the Americas, Charlestown, KNA

**Keywords:** men who have sex with other men (msm), hiv, neurosyphilis, sepsis, respiratory failure

## Abstract

This case report presents the case of a 58-year-old Caucasian male with hypercapnic respiratory failure (type 2 respiratory failure) and septic shock attributed to pneumonia. He also had multiorgan dysfunction and was subsequently diagnosed with neurosyphilis in the setting of underlying HIV. The patient initially presented with worsening shortness of breath and bilateral lower extremity edema. Further evaluation revealed HIV positivity with immunosuppression. The presence of neurologic symptoms on physical examination prompted investigation for an alternative etiology, ultimately leading to the diagnosis of neurosyphilis.

## Introduction

Neurosyphilis, caused by *Treponema pallidum*, can manifest at any time following the initial infection. The incidence of syphilis has been steadily increasing, particularly among men who have sex with men (MSM) [1,2]. Syphilis is known as “the great imitator” due to its variable clinical presentation and diagnostic challenges [1]. This case report highlights a patient diagnosed with neurosyphilis in the setting of underlying HIV infection who presented with type 2 respiratory failure and septic shock [3-6]. This case report highlights the significance of considering neurosyphilis as a potential cause for unexplained neurological symptoms, particularly in patients who have risk factors such as HIV infection, immunosuppression, and MSM.

## Case presentation

A 58-year-old Caucasian male with a smoking history of 22 pack-years and no known past medical issues presented with a three-month history of shortness of breath and bilateral lower extremity edema. His primary complaint was shortness of breath. The patient exhibited tachypnea with a respiratory rate of 35-40 breaths per minute. His vital signs were otherwise within normal limits. His Glasgow Coma Scale score was 15 but he quickly deteriorated and became drowsy and lethargic. Initially, he was administered 15 L of oxygen via a non-rebreather mask, but due to worsening hypercapnic respiratory failure, he was transferred to the intensive care unit and intubated.

Upon arrival at the emergency department, his venous blood gas analysis revealed a pH of 7.18, pCO_2_ of 84 mmHg, pO_2_ of 40 mmHg, and HCO_3_ of 31 mg/L. An electrocardiogram indicated atrial flutter with rapid ventricular response at a rate of 177/minute. His heart rate was controlled using a cardizem drip, adenosine, lopressor, and amiodarone. An echocardiogram demonstrated an ejection fraction of 55% with normal valve function, as well as elevated pulmonary arterial pressures. Upon admission, his troponin I level was 0.332 ng/mL, which gradually decreased and was likely elevated due to demand ischemia. Further laboratory results included a procalcitonin level of 2.38 ng/mL, B-type natriuretic peptide of 879 pg/mL, sodium level of 147 mmol/L, blood urea nitrogen of 57 mg/dL, creatinine of 2.87 mg/dL, alkaline phosphatase of 153 U/L, alanine transaminase of 3,650 U/L, aspartate transaminase of 5,329 U/L, total protein of 4.6 g/dL, albumin of 2.1 g/dL, total bilirubin of 2.1 mg/dL, and white blood cell count (WBC) of 12.5 × 10^9^/L. Gamma-glutamyl transpeptidase, activated partial thromboplastin time, and international normalized ratio were within normal limits, and there were no concerns for bleeding. The remaining comprehensive metabolic panel and complete blood count were within normal limits unless mentioned otherwise. The pneumonia severity index was calculated at 158, class V, corresponding to a 27% mortality rate, thus recommending hospitalization based on risk. The patient did not require vasopressors or inotropes.

A chest X-ray at the time indicated an infiltrate in the right lower lobe of the lung. The CT scan of the chest without contrast revealed a small right pleural effusion, along with mild pneumonia, mild atelectasis, and consolidation within the right lower lobe. Laboratory results and imaging indicated that the patient was experiencing volume overload, septic shock, and lobular pneumonia. Physical examination revealed wheezing in the upper left lung field. The left radial pulse was not palpable at the time, and both upper and lower extremities were edematous and cool to the touch. Hepatomegaly was observed with the liver margin percussed 5 cm below the costal margin. Biceps and patellar reflexes were 3+ bilaterally, with left patellar clonus and bilateral ankle clonus exceeding 10 beats. Additionally, the patient experienced unprovoked spasming of his right leg for approximately three seconds. Broad-spectrum antibiotics, vancomycin, and cefepime were initiated, and on day five, the patient was transitioned to Rocephin after isolating* Streptococcus pneumoniae* from bronchoalveolar lavage and obtaining sensitivities.

Electroencephalography findings suggested mild encephalopathy without evidence of seizures. The presence of neurological symptoms such as hyperreflexia and clonus, along with the patient’s high-risk status for HIV and syphilis, prompted further evaluation. Considering the patient’s high-risk sexual practices (MSM), HIV testing was conducted, which revealed HIV positivity, immunosuppression, and a reactive rapid plasma reagin (RPR) and fluorescent treponemal antibody consistent with syphilis. A lumbar puncture confirmed the diagnosis of neurosyphilis with a reactive Venereal Disease Research Laboratory test. The lumbar puncture was performed, revealing 13 WBC/mm^3^, two red blood cells/mm^3^, a glucose level of 57 mg/dL, and a protein level of 35 mg/dL. The patient had an absolute CD4 count of 135 cells/mL and a CD4 percentage of 31%. HIV-1 quantitative nucleic acid amplification test showed a viral load of 2,683 copies/mL, and the RPR test was reactive with a quantitative RPR of 1:512. Testing for all other bacterial, viral, fungal, and yeast infections yielded negative results. Intravenous (IV) penicillin G was administered for two weeks (IV penicillin G Q4 4M units), resulting in the resolution of neurological symptoms. On day 10 of hospitalization, the patient’s arterial blood gas analysis indicated a pH of 7.46, pCO_2_ of 53 mmHg, pO_2_ of 62 mmHg, and HCO_3_ of 37 mg/L, and he was successfully extubated.

After completing a 14-day course of IV penicillin G in the hospital, the patient faced difficulty finding a skilled nursing facility that would accept them. Due to the recent HIV diagnosis, it was decided that the patient would follow up as an outpatient with an infectious disease doctor to initiate antiretroviral therapy and preemptive antibiotics per their CD4 counts. The patient was stable at the time of discharge.

## Discussion

Neurosyphilis is a complication that can occur at any stage following *Treponema pallidum* infection. With increasing rates of syphilis, clinicians should expect to encounter more cases, particularly among high-risk populations such as MSM and individuals with HIV infection. The rates of syphilis have steadily increased since the early 2000s reaching a new high of 176,713 cases in 2021 [1]. The presentation of neurosyphilis can vary widely and routine screening should be considered in MSM and persons with HIV to prevent an emerging syphilis epidemic. This case report emphasizes the need for early detection of neurosyphilis through symptomatology recognition to improve clinical outcomes [2-6]. Table 1 demonstrates how variable the symptomatology and clinical presentation can be and how neurosyphilis can present in various forms, such as meningovascular, tabes dorsalis, general paresis, and asymptomatic neurosyphilis [4,6]. The symptoms may range from mild cognitive impairments to severe neurological deficits, depending on the stage of the disease [2].

**Table 1:** Manifestations of neurosyphilis. [3,4]. CSF = cerebrospinal fluid

Asymptomatic neurosyphilis	Patients have CSF abnormalities without any neurological symptoms
Meningeal	Inflammation of the meninges. Presents with headaches, nausea, vomiting, neck stiffness, photophobia, cranial nerve deficits, and seizures
Meningovascular	Inflammation of the meninges + endarteritis causing thrombosis and infarction of the cerebral tissue and/or spinal vessels. Presentation depends on the site of vascular insult
General paresis	Chronic meningoencephalitis resulting in cerebral atrophy. Presents with mood changes (personality changes, lability, memory and judgment impairment, delusions, and seizures); psychiatric disorders including depression, mania, and psychosis; pupillary abnormalities; dysarthria; and tremors
Tabes dorsalis	Due to degeneration of the dorsal column/nerve roots. Presents with ataxia, bladder dysfunction, paresthesia, Charcot’s joints, pupillary changes (Argyll Robertson pupil), and optic atrophy

Neurosyphilis occurs when the *Treponema pallidum* bacterium invades the central nervous system. When neurosyphilis presents with concomitant respiratory failure, this poses several challenges for healthcare professionals. Diagnosing neurosyphilis with accompanying respiratory failure requires a high level of clinical suspicion. This is because many patients do not exhibit the typical signs of syphilis that are predominately taught in medical schools, such as genital ulcers or rashes [2]. The mechanism of respiratory failure can be secondary to nerve involvement, causing subsequent muscle weakness or paralysis. Due to the severity of this sexually transmitted infection, timely recognition and diagnosis is imperative. This is an important consideration when encountering a patient with syphilis and concurrent respiratory failure. However, in this specific case, the patient’s respiratory failure occurred in the context of concomitant lobar pneumonia. Furthermore, when managing patients with neurosyphilis and respiratory failure, there are important treatment considerations. It is crucial to determine whether the patient’s respiratory failure is a result of syphilis or another underlying cause.

The treatment for all forms of neurosyphilis is based on parenteral penicillin. In cases of concomitant respiratory failure, close monitoring and airway protection are pivotal in managing patients with both conditions simultaneously. In this specific case, the patient required antibiotics to treat the underlying pneumonia. This patient presented with hyperreflexia and clonus, which are symptoms associated with general paresis. The long-term prognosis of patients with neurosyphilis and concomitant respiratory failure is poor and may increase the burden of the existing disease and mortality [7].

## Conclusions

This case report underscores the challenges in diagnosing and managing multiple comorbidities in an acute setting. Timely recognition of neurosyphilis can lead to better clinical outcomes, especially in patients with multiple comorbidities. Clinicians should maintain a high index of suspicion for neurosyphilis in patients presenting with unexplained neurological symptoms, particularly in populations disproportionately affected by the disease such as in the MSM population. Due to the variability of symptoms in the clinical presentation of neurosyphilis, medical professionals should have a high level of suspicion for neurosyphilis. This case report highlights the importance of recognizing the numerous presenting symptoms of neurosyphilis and understanding how respiratory failure could potentially play a role. Neurosyphilis and acute hypoxic respiratory failure are the potentially life-threatening complications of syphilis.
